# Stage-Dependent Brain Plasticity Induced by Long-Term Endurance Training: A Longitudinal Neuroimaging Study

**DOI:** 10.3390/life15091342

**Published:** 2025-08-25

**Authors:** Keying Zhang, Qing Yan, Ling Jiang, Dongxue Liang, Chunmei Cao, Dong Zhang

**Affiliations:** 1 Department of Physical Education, Southeast University, Nanjing 210096, China; bsuzky0812@163.com (K.Z.); l1ng_jiang@163.com (L.J.); 2 Institute of Artificial Intelligence in Sports, Capital University of Physical Education and Sports, Beijing 100191, China; 13181579677@163.com (Q.Y.); liangdongxue@cupes.edu.cn (D.L.); 3 Division of Sports Science and Physical Education Tsinghua University, Tsinghua University, Beijing 100084, China; caocm@tsinghua.edu.cn

**Keywords:** endurance training, brain plasticity, resting-state fMRI, functional reorganization, college student

## Abstract

Long-term physical training is known to induce brain plasticity, yet how these neural adaptations evolve across different stages of training remains underexplored. This two-year longitudinal study investigated the stage-dependent effects of endurance running on brain structure and resting-state function in healthy college students. Thirty participants were recruited into three groups based on their endurance training level: high-level runners, moderate-level runners, and sedentary controls. All participants underwent baseline and two-year follow-up MRI scans, including T1-weighted structural imaging and resting-state fMRI. The results revealed that the high-level runners exhibited a significant increase in degree centrality (DC) in the left dorsolateral prefrontal cortex (DLPFC). In the moderate-level group, more widespread changes were observed, including increased gray matter volume (GMV) in bilateral prefrontal cortices, medial frontal regions, the right insula, the right putamen, and the right temporo-parieto-occipital junction, along with decreased GMV in the posterior cerebellum. Additionally, DC decreased in the left thalamus and increased in the right temporal lobe and bilateral DLPFC; the fractional amplitude of low-frequency fluctuations (fALFF) in the right precentral gyrus was also elevated. These brain regions are involved in executive control, sensorimotor integration, and motor coordination, which may suggest potential functional implications for cognitive and motor performance; however, such interpretations should be viewed cautiously given the modest sample size and study duration. No significant changes were found in the control group. These findings demonstrate that long-term endurance training induces distinct patterns of brain plasticity at different training stages, with more prominent and widespread changes occurring during earlier phases of training.

## 1. Introduction

Recent advances in neuroimaging have significantly enhanced our understanding of brain plasticity in response to long-term endurance training. Cross-sectional studies have demonstrated that individuals engaged in prolonged aerobic exercise exhibit distinct structural and functional brain features, including increased gray matter volume (GMV), enhanced functional connectivity (FC), and an increased fractional amplitude of low-frequency fluctuations (fALFF) in task-relevant regions. For instance, Herting et al. (2014) found that adolescent males with higher aerobic fitness showed greater streamline counts in white matter tracts associated with frontal and motor processing, such as the corticospinal tract and anterior corpus callosum, suggesting enhanced structural connectivity in networks supporting executive and motor control [1]. Raichlen et al. (2016) observed that collegiate endurance runners exhibited stronger FC between the frontoparietal network and regions involved in executive functions, along with enhanced anti-correlations between the default mode network (DMN) and motor/sensory areas—patterns associated with better cognitive control and motor coordination [2]. Moreover, our own prior work demonstrated that endurance athletes at varying levels showed distinctive patterns of brain plasticity. High-level runners exhibited greater GMV and functional indicators in regions related to memory, motor control, and sensory integration, while moderate-level runners showed pronounced reorganization in frontal and DMN regions [3]. While these cross-sectional findings are valuable for capturing long-term adaptations in athletes, they are inherently limited in their ability to infer causality, leaving it unclear whether the observed neural differences stem from endurance training itself or from pre-existing traits that predispose individuals to excel in aerobic activities. In contrast, longitudinal designs track within-subject changes over time, establish a temporal sequence between training and neural outcomes, and reduce inter-individual variability, thereby providing stronger, though not definitive, evidence for causality [4,5]. Therefore, longitudinal studies are needed to directly assess the neural impact of sustained endurance training.

The existing longitudinal studies on aerobic exercise-induced neuroplasticity have primarily focused on children, the elderly, and clinical groups. For example, in 2024, Qu et al. investigated functional activation and working memory in deaf children [6]. Colcombe and colleagues demonstrated associations between aerobic fitness and brain plasticity in older adults [7]; however, such associations, particularly from cross-sectional studies, suggest relationships rather than definitive causal effects. In 2021, Yogev-Seligmann et al. reported that improved functional plasticity in individuals with amnestic mild cognitive impairment was mediated by improvements in cardiorespiratory fitness [8]. In contrast, longitudinal research examining the impact of sustained endurance exercise on brain structure and function in healthy young adults, particularly college-aged individuals, is still insufficient. This gap is particularly significant given that young adulthood represents a critical period of late neurodevelopmental maturation, which is characterized by heightened structural and functional brain plasticity, offering unique potential for adaptive change. Furthermore, the establishment of exercise habits during this formative life stage is pivotal for long-term cognitive and physical health trajectories. Consequently, elucidating the neuroplastic mechanisms underlying long-term endurance training in this understudied population may hold substantial theoretical importance and practical relevance. Mechanistically, long-term aerobic training may enhance brain plasticity through multi-level processes [9]. Specifically, at the molecular level, it elevates neurotrophic factors such as BDNF, IGF-1, and VEGF, which support neural health. At the cellular level, these factors promote neurogenesis, synaptogenesis, and angiogenesis. Such changes can reorganize the brain structure and connectivity at the systems level, ultimately improving cognitive and motor performance [9].

Motor skill acquisition is widely understood through the classic three-stage model proposed by Fitts and Posner [10,11], which describes a progression from the cognitive stage, through the associative stage, to the autonomous stage. Each stage reflects distinct behavioral characteristics and underlying neural processes. In the early cognitive stage, individuals rely heavily on attention and conscious control, typically involving broad activation across multiple brain regions. As learning progresses, neural processing becomes more focused and efficient, aligning with the Neural Efficiency Hypothesis (NEH) [12,13]. This model provides a valuable framework for understanding how the brain adapts to physical training over time. From a neuroscience perspective, such stage-based transitions imply that brain plasticity is not uniform but evolves with training experience. However, the specific characteristics of neuroplasticity changes across different stages of physical training—particularly in endurance sports—remain poorly defined. Most existing research focuses on expert–novice comparisons using cross-sectional designs, which overlook the dynamic and transitional nature of brain adaptations. Clarifying how brain structure and function reorganize at each training stage is essential for both theoretical advancement and practical application. A deeper understanding of stage-dependent plasticity could inform the development of personalized training strategies, enabling more effective progression through the stages of motor learning and optimizing both cognitive and physical outcomes.

Given the identified research gaps, this longitudinal investigation examined the effects of long-term sustained endurance training on brain structure and resting-state functional activity in healthy young adult university students. Here, ‘long-term’ refers specifically to the 2-year supervised training period in this longitudinal design. This research presents two primary innovations: (1) the adoption of a longitudinal design to establish causal relationships between endurance training and neuroplasticity changes in brain structure and function, building upon prior cross-sectional evidence; and (2) the investigation of the stage-dependent characteristics of endurance training-induced neuroplasticity. Specifically, it is the first study to explicitly compare neural adaptation mechanisms across distinct endurance levels (high-level, moderate-level, and control groups) during long-term training, with a focused inquiry into whether neuroplasticity is maximized in moderate-level endurance athletes. Based on the theoretical framework of stage-dependent plasticity, the study proposed the following hypotheses: (1) long-term endurance training induces neuroplasticity changes that have stage-dependent characteristics; and (2) moderate-level endurance runners will have the most pronounced neuroplasticity benefits, whereas high-level athletes with exhibit relatively stable neural adaptations with limited further plasticity despite increased training loads. This expectation is grounded in the notion that, at moderate levels of training, athletes may still be in a phase of heightened neural adaptability, whereas at high levels, their neural systems may have already undergone substantial reorganization, leading to a plateau effect in further plastic changes.

## 2. Materials and Methods

### 2.1. Participants

This study adopted a prospective longitudinal design to investigate the effects of long-term endurance training on brain structure and resting-state functional plasticity. A total of thirty participants were recruited, who were placed into a high-level endurance athlete group, a moderate-level endurance runner group, or a non-athlete healthy control group. The high-level group consisted of athletes meeting the Chinese National First-Class Athlete standard, which represents a nationally certified performance benchmark. The moderate-level group included amateur runners who trained regularly in endurance running (≥3 sessions per week) but did not meet the criteria for national classification. The control group comprised sedentary individuals without regular endurance training. From this perspective, the three groups naturally represent three distinct stages of endurance training: advanced (high-level athletes), intermediate (moderate-level runners), and untrained (controls). All the initially recruited participants (*N* = 30) completed baseline MRI scans. Although 8 participants withdrew before the follow-up, their baseline data were included in cross-sectional group comparisons. The baseline demographic and training characteristics for the three groups are summarized in Table 1.

**Figure 1:**
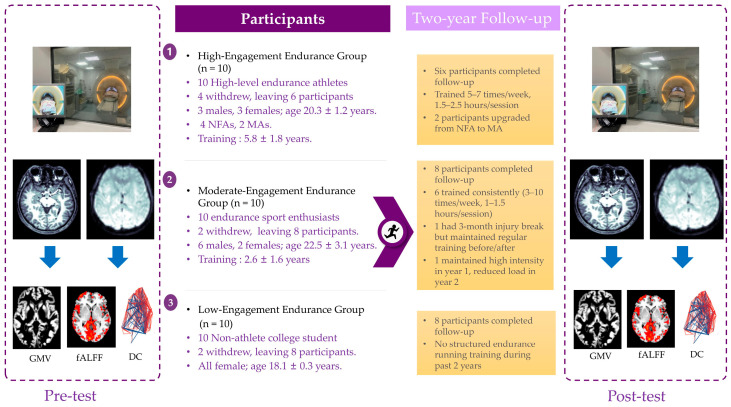
Experimental procedure flowchart (longitudinal study design). Flowchart depicts group allocation, baseline/post-test MRI sessions, and key outcome measures (GMV/fALFF/DC). Arrows indicate 2-year endurance training period. Participants were first divided into three groups and completed a baseline MRI scan, and then underwent weekly training at different intensities over two years, followed by a post-training MRI scan. NFA: National First-Class Athlete; MA: Master Athlete; GMV: gray matter volume; fALFF: fractional amplitude of low-frequency fluctuations; DC: degree centrality.

The inclusion criteria were as follows: (a) age between 17 and 30 years; (b) right-handed; (c) normal or corrected-to-normal vision; (d) no history of cardiovascular, musculoskeletal, or neurological disorders; and (e) ability to safely complete MRI scanning procedures. The exclusion criteria included the following: (a) clinically diagnosed neurological disorder (e.g., epilepsy or stroke history); (b) active musculoskeletal condition or traumatic injury affecting mobility; or (c) significant systemic disease (cardiovascular, metabolic, or autoimmune disorder). The initial recruitment yielded 10 high-level long-distance runners, from which, 4 withdrew due to injury or academic commitments, resulting in a final cohort of 6 athletes (3 males/3 females; age: 20.3 ± 1.2 years; 4 National First-Class Athletes and 2 Master Athletes; mean training history: 5.8 ± 1.8 years). Similarly, 10 moderate-level runners were initially enrolled, with 2 withdrawing for academic reasons, leaving 8 participants (6 males/2 females; age: 22.5 ± 3.1 years; training history: 2.6 ± 1.6 years). The healthy control group consisted of 10 university students, 2 of whom withdrew due to academic obligations, yielding 8 controls (all female; age: 18.1 ± 0.3 years). All the retained participants (N = 22) were right-handed. The participants completed baseline MRI scans, and then engaged in group-specific endurance training regimens for two years, followed by a post-training MRI assessment (see Figure 1 for experimental workflow).

### 2.2. Image Data

Brain imaging data were acquired using a Philips 3.0 T MRI scanner equipped with a standard 32-channel head coil. Structural images were acquired using a T1-weighted sequence to assess gray matter morphology. The parameters were flip angle = 8°, number of slices = 180, slice thickness = 1 mm, acquisition matrix = 80 × 80, and FOV = 230 × 230 mm^2^. At the same time, resting-state functional images were collected using a single-shot echo-planar imaging (EPI) sequence to measure BOLD signals. The scanning parameters were as follows: repetition time (TR) = 2000 ms, echo time (TE) = 30 ms, flip angle = 90°, number of slices = 37, slice thickness = 3 mm, acquisition matrix = 80 × 80, field of view (FOV) = 230 × 230 mm^2^, and voxel size = 2.87 × 2.87 × 3.50 mm^3^. During scanning, the participants were instructed to “keep their eyes closed, stay relaxed, remain awake, and try not to think about anything specific.” To reduce the auditory and psychological discomfort caused by the loud gradient switching noise, earplugs and noise-canceling headphones were provided. Head motion was strictly controlled during the experiment, as it can introduce artifacts that are difficult to fully correct in post-processing. The participants were explicitly instructed to “remain as still as possible throughout the entire scan,” and foam padding was inserted between the participant’s head and the coil to minimize head movement.

Each participant underwent MRI scans at two time points: pre-training and post- training. After the initial scan, no training or behavioral intervention was administered. The participants were simply informed that they would be invited to complete a follow-up in the future. Two years later, the participants underwent a second MRI scan. The participants completed a retrospective physical activity questionnaire, which was specifically designed for this study that drew on the general structure of widely used physical activity surveys reported in previously published literature. The questionnaire included (1) an overall evaluation of whether the participants had consistently exercised over the past two years; (2) their preferred sports or exercise modalities; (3) retrospective estimates of training frequency and duration over the past six months and the past week; and (4) self-reported sleep patterns. For the high-level athletes, the self-reported data were cross-checked against training plans and actual training logs; for the moderate-level runners, they were verified against training schedules and attendance records. This combination of subjective reports and objective documentation was used to ensure accurate classification of training exposure.

### 2.3. Image Data Processing

Image data processing was conducted with reference to our previously published work [3]. Resting-state fMRI data were preprocessed using RESTplus V1.2 [14]. The preprocessing steps included removal of the first five volumes, slice timing correction, realignment for head motion, co-registration with individual T1-weighted anatomical images, spatial normalization to the MNI space, and spatial smoothing using a Gaussian kernel. To reduce physiological and motion-related noise, nuisance covariates including white matter and cerebrospinal fluid signals, as well as 24 Friston head motion parameters, were regressed out. For assessment of structural plasticity, T1-weighted anatomical images were processed using the VBM8 toolbox. The images were segmented into gray matter, white matter, and cerebrospinal fluid, which were normalized to MNI space using the DARTEL algorithm and smoothed with an 8 mm full width at half maximum (FWHM) Gaussian kernel. This procedure yielded whole-brain GMV maps for each participant.

Resting-state functional plasticity was evaluated using two commonly applied indices. fALFF was calculated to reflect the relative power of spontaneous neural activity within the low-frequency band (0.01–0.1 Hz). DC was computed to assess the functional integration of each voxel within the whole-brain network, with the correlation threshold set at r = 0.25. Both fALFF and DC maps were transformed using Fisher’s z-transformation for statistical analysis. All MRI measures (GMV, fALFF, and DC) demonstrate good test–retest reliability in longitudinal designs. The reliability of these data has been verified by multiple peer-reviewed studies [15,16].

### 2.4. Statistical Analysis

Statistical analysis of the preprocessed neuroimaging data was performed using SPM12 (http://www.fil.ion.ucl.ac.uk/spm), accessed between 1 November 2019, and 30 October 2021. Paired-sample *t*-tests were conducted within each participant group to examine the longitudinal changes in GMV, fALFF, and DC between baseline and the 2-year follow-up, thereby assessing the structural and functional brain reorganization over this interval. This approach was chosen to align with our primary aim of detecting stage-specific longitudinal changes. Thus, we prioritized within-stage comparisons to characterize these potential differences. The analyses employed a voxel-level threshold of *p* < 0.001 (uncorrected) with family-wise error (FWE) correction for whole-brain multiple comparisons, while age was incorporated as a covariate in all statistical models. The results were subsequently tabulated and visualized in MNI standard space using BrainNet Viewer_20181219.

## 3. Results

### 3.1. Participant Retention and Training Adherence over the Follow-Up Period

In the high-level endurance group, four participants dropped out due to injury or academic demands, leaving six who completed the two-year follow-up. According to the retrospective questionnaire, all six reported consistent endurance training throughout the follow-up period. Two participants reported occasional injuries, while the remaining four remained injury-free. Their weekly training frequency ranged from five to seven sessions, with each session lasting 1.5 to 2.5 h. Notably, two participants were promoted from first-level national athletes to elite-level expert during this period.

In the moderate-level endurance group, two participants withdrew due to academic reasons, leaving eight who completed the follow-up. Among them, six reported regular endurance training over the past two years, with training frequencies ranging from three to ten sessions per week and each session lasting 1 to 1.5 h. One participant reported a three-month break from training due to injury but maintained regular running before and after. Another participant maintained high-intensity training during the first year but reported a significant reduction in training load during the second year.

In the control group, two participants withdrew due to academic demands, with eight completing the follow-up. All eight controls reported no structured endurance training.

The baseline cross-sectional comparisons revealed significant group differences in training history (F = 98.7, *p* < 0.001) and BMI (F = 3.6, *p* = 0.04). The post hoc tests confirmed longer training in the high-level (6.3 ± 1.6) vs. moderate-level runners (2.6 ± 1.0, *p* < 0.001), while the controls had no training (0 ± 0). BMI was higher in the moderate-level runners (21.7 ± 1.8) than in the controls (20.2 ± 2.1, *p* = 0.03), but the high-level runners (20.4 ± 1.3) did not differ from either group (*p* > 0.05).

### 3.2. Brain Plasticity in High-Level Endurance Runners

**Figure 2:**
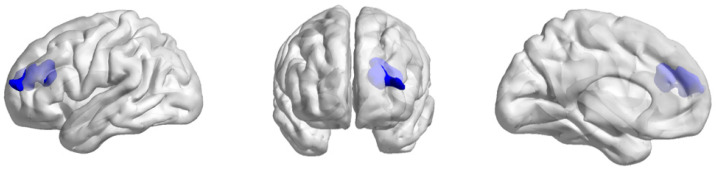
DC changes in the left DLPFC. Blue indicates regions where DC values were significantly higher post-training than pre-training (increase). No significant decreases were observed in this group.

The paired-sample *t*-test results showed a significant increase in DC in the DLPFC in the high-level endurance group after two years of professional endurance training (*p* < 0.001, FWE corrected; see Table 2 and Figure 2). However, the changes in GMV and fALFF in this region did not survive multiple comparison correction at *p* < 0.001, and no significant differences were observed.

### 3.3. Brain Plasticity in Moderate-Level Endurance Runners

Two years of systematic endurance training at the amateur level induced widespread and significant changes in both structural and functional brain plasticity in the moderately trained long-distance runners.

**Figure 3:**
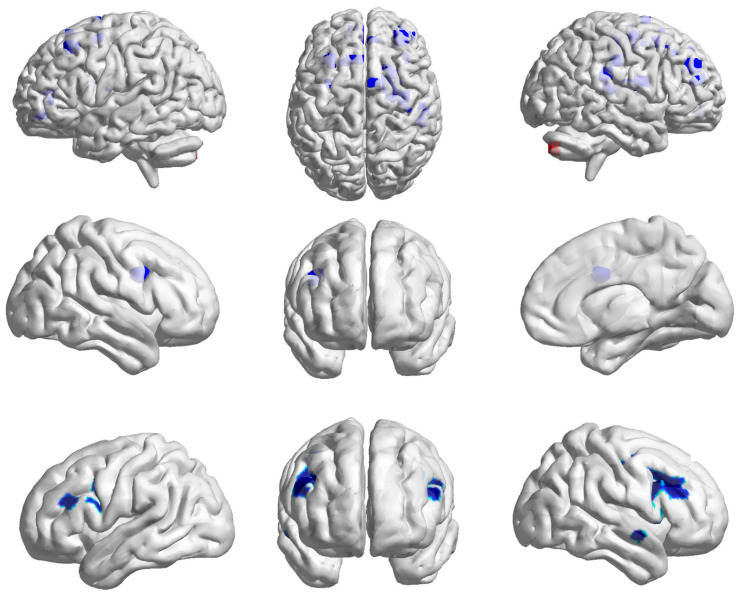
Brain plasticity over two years. From top to bottom: GMV, fALFF, and DC. GMV: gray matter volume; fALFF: fractional amplitude of low-frequency fluctuations; DC: degree centrality; DLPFC: left dorsolateral prefrontal cortex. Blue indicates regions where values were significantly higher post-training than pre-training (increase). Red indicates regions where values were significantly lower post-training than pre-training (decrease).

The paired-sample *t*-test results revealed significant decreases in GMV in the bilateral posterior cerebellum, alongside significant GMV increases in multiple regions including the bilateral prefrontal cortex (such as the dorsolateral prefrontal cortex, premotor cortex, and precentral gyrus), bilateral medial frontal cortex, right insula, right putamen, and the right fronto-parieto-occipito-temporal junction (*p* < 0.001, FWE corrected; see Table 3 and Figure 3).

In addition, the DC of the left thalamus was significantly reduced, while DC values in the right temporal lobe and bilateral dorsolateral prefrontal cortex were significantly increased (*p* < 0.001, FWE corrected; see Table 2 and Figure 3). A significant increase in fALFF was also observed in the right medial frontal cortex (*p* < 0.001, FWE corrected; see Table 3 and Figure 2).

### 3.4. Longitudinal Brain Changes in the Control Group

The paired-sample *t*-tests revealed no significant changes in GMV, fALFF, or DC in this group. Under the same multiple comparison correction threshold applied to the other two groups, no brain regions exhibited significant neuroplasticity changes across the two-year period.

## 4. Discussion

Although endurance exercise is known to benefit brain health, previous neuroimaging studies have mainly compared novices with elite athletes, leaving a gap in under-standing how neural adaptations unfold at intermediate training stages. Identifying the stage at which training yields the greatest neural benefits may be clinically relevant for optimizing athletic training and informing neuro-rehabilitation strategies. To address this gap, we adopted a longitudinal design to investigate stage-dependent plasticity in endurance runners of different performance levels. This study aimed to examine the changes in brain structure and resting-state functional activity before and after a two-year period of endurance training. The results showed that, following two years of professional training, the high-level endurance group exhibited a significant increase in DC in the DLPFC. In contrast, the moderate-level group demonstrated more widespread plasticity-related changes, including alterations in both structural and functional plasticity across multiple brain regions, such as the prefrontal cortex, temporal lobe, insula, and posterior cerebellum. No significant structural or functional changes were observed in the sedentary control group during the same period. These findings support a stage-dependent effect of endurance training, with the intermediate stage showing the greatest neural adaptation in both brain structure and function. This pattern aligns with our theoretical proposal, grounded in the three-stage model of motor skill acquisition, which suggests that neural recruitment and reorganization differ across stages of expertise. By mapping these adaptations longitudinally, our results provide empirical evidence for stage-dependent neural plasticity and have implications for optimizing training and rehabilitation programs.

### 4.1. Neural Mechanisms of Running-Induced Plasticity

#### 4.1.1. Frontal Cortex Plasticity

Our findings demonstrate that long-term endurance training significantly influences the structural and functional plasticity of the frontal cortex, a region integral to episodic memory, spatial attention, motor regulation, action execution, rhythm coordination, and sensorimotor integration. The high-level endurance runners showed increased GMV, primarily in the dorsolateral prefrontal cortex (DLPFC), whereas the moderate-level runners exhibited broader GMV enhancements across several frontal sub-regions, including the DLPFC, premotor cortex, precentral gyrus, and medial frontal areas.

Traditionally, the frontal cortex is recognized for its role in cognitive processing; however, recent evidence is increasingly highlighting its substantial involvement in motor control. Numerous studies indicate that the frontal cortex is crucial for episodic memory retrieval [17,18,19] and serves as a central node in modulating spatial attentional resource allocation [20]. Notably, physical exercise has been consistently reported to induce enduring frontal cortical plasticity [21]. For instance, longitudinal research showed greater prefrontal cortical thickness in individuals who maintained physical activity for over 21 years [22]. Similarly, older adults who engaged in triathlon training exhibited increased medial prefrontal cortex (mPFC) and precentral gyrus thickness compared to sedentary controls [23]. Even short-term interventions, such as a 6-week Nintendo Wii fitness program, resulted in increased GMV in the DLPFC, posterior cingulate cortex/precuneus, hand motor area, occipital cortex, and cerebellum among elderly participants [24].

The frontal cortex participates in several critical brain networks, including the frontoparietal control network, DMN, salience network, and executive control network. Each frontal sub-region uniquely contributes to motor and cognitive functions: for example, the DLPFC, premotor cortex, supplementary motor area, and orbitofrontal cortex have distinct roles in action control. Specifically, the DLPFC and posterior parietal cortex constitute the executive control network, which is associated with high-level executive and cognitive functions [25]. The cingulo-opercular network (CON), formed by connections between the prefrontal cortex, dorsal anterior cingulate cortex, inferior parietal lobe, basal ganglia, and thalamus, along with the salience network (ventrolateral prefrontal cortex, anterior insula, and dorsal anterior cingulate cortex), primarily directs attentional control and error monitoring during motor tasks [26,27,28]. Moreover, the premotor cortex is closely involved in sequencing motor skills and rhythm coordination. Increased GMV in this region has been reported in golfers [21,29], with elevated premotor cortex activation during motor imagery tasks [30]. Structural modifications in premotor areas have also been noted among divers [31] and ballet dancers [32].

In addition, the mPFC, together with the precuneus, posterior cingulate cortex, and inferior parietal lobule, comprises the DMN, which is frequently activated during tasks involving spontaneous cognition [33,34,35,36]. The mPFC, a core component of the DMN, is notably involved in self-referential episodic memory [37]. Previous research found increased DMN activity in basketball players, which is potentially linked to frequent self-relevant episodic memory processing during games [38]. Exercise interventions also appeared to have enhanced DMN FC in older adults [39]. Collectively, these findings underscore that long-term endurance running may promote structural and functional adaptations within the frontal areas critical for cognitive–motor integration, spatial attention, and episodic memory.

#### 4.1.2. Functional Reorganization of Sensorimotor Regions

Endurance training may have led to notable functional reorganization, as evidenced by the increased DC in the left DLPFC for both the moderate- and high-level runners, suggesting structural and functional enhancement in this key area. Furthermore, the precentral gyrus, or primary motor cortex (M1), which is essential for consolidating and accurately executing learned motor skills [40,41], exhibited increased GMV and density with prolonged training [42,43]. Notably, elite gymnasts showed higher nodal degree and regional efficiency in the left precentral gyrus white matter network compared to controls [44]. Similarly, dancers demonstrated higher local consistency and fALFF in bilateral precentral gyri compared to non-dancers, along with enhanced FC between bilateral pre- and postcentral gyri [45]. Our results indicated significant fALFF elevation in the right precentral gyrus among the moderate-level endurance runners following two years of training. Collectively, this suggests that long-term endurance training may enhance brain regions integral to cognitive and sensorimotor integration.

Additionally, we observed increased GMV in the right insula, a region crucially involved in motor learning [46]. The insula is strongly coupled with the premotor, sensorimotor, supplementary motor areas, and cingulate cortex, playing a pivotal role in sensorimotor integration [47]. Although a previous study reported decreased left insular GMV after long-term aerobic dance in young adults, these results lacked rigorous multiple-comparison corrections [48]. Hence, our findings support that endurance training may play a beneficial role in enhancing insular structural integrity, which is associated with motor learning and sensorimotor integration.

#### 4.1.3. Cerebellar Plasticity and Efficiency

Intriguingly, we found decreased GMV in the posterior cerebellar lobules among the moderate-level runners after two years of training, a finding best explained by the neural efficiency hypothesis. According to this hypothesis, long-term motor practice induces selective, streamlined cortical activation patterns, reducing unnecessary neural recruitment and optimizing central resources [49]. Given the cerebellum’s prominent role in motor generation, control, and regulation, prolonged endurance training may have strengthened synaptic pruning and dendritic spine formation, enhancing neural efficiency so that the same motor output could be achieved with reduced cerebellar GMV.

#### 4.1.4. Hippocampal Stability: Selection or Threshold Effect?

Notably, our study did not reveal structural or functional changes in the hippocampus or parahippocampal regions. This was somewhat unexpected, as numerous previous studies have demonstrated a close association between aerobic exercise and hippocampal as well as parahippocampal plasticity [50,51]. Our own prior research also showed structural and functional reorganization in these regions among aerobic-trained athletes [3]. Several explanations exist for this observation. Firstly, it is possible that two years of high-intensity endurance training may not induce hippocampal changes in already trained athletes, suggesting that the previously observed larger hippocampal volumes among high-level endurance runners might be innate characteristics rather than training-induced adaptations, thus serving as potential selection criteria. However, aerobic exercise is generally recognized to stimulate hippocampal dentate gyrus neurogenesis, enhance neuronal survival [52], and elevate hippocampal brain-derived neurotrophic factor (BDNF), insulin-like growth factor, and vascular endothelial growth factor levels [52,53,54]. Several animal and human studies have reported increased hippocampal volumes following aerobic exercise [55], reinforcing the importance of aerobic exercise for hippocampal function.

Secondly, our stringent statistical approach (FWE-corrected threshold at *p* < 0.001) combined with the limited sample size may have elevated the risk of type II errors. Nevertheless, re-analysis at a relaxed threshold (*p* < 0.01) still revealed no hippocampal or parahippocampal differences, partially mitigating this concern.

Lastly, the absence of hippocampal adaptations might be attributed to excessively high training loads and intensities exceeding the optimal neuroplasticity thresholds. A previous review highlighted that training intensity, frequency, and modality significantly influence hippocampal neurogenesis, with moderate intensities being protective, whereas excessive intensities may divert blood flow to skeletal muscles, thereby reducing cerebral perfusion and promoting neuronal apoptosis [56]. Given that our participants engaged exclusively in long-distance running with high training volumes, these factors may have contributed to the lack of detectable hippocampal plasticity. Therefore, hippocampal GMV remains a potential indicator for identifying endurance talent rather than reflecting training-induced adaptations within elite populations.

### 4.2. Beyond Cross-Sectional Findings: A Longitudinal Confirmation of Causality

Our previous cross-sectional research employing the expert–novice paradigm provided initial evidence that endurance athletes exhibit distinct structural and functional brain characteristics depending on their training levels. Specifically, high-level endurance athletes showed greater GMV and enhanced functional activity in brain regions associated with memory consolidation, motor control, and sensory processing compared to moderate-level athletes and sedentary controls. However, such cross-sectional designs inherently limit our ability to infer causality as the observed neural differences might reflect pre-existing individual variations rather than training-induced adaptations. To overcome this limitation, the current longitudinal study tracked brain plasticity changes over two years in groups categorized by distinct endurance training intensities. Our findings confirmed and provide preliminary evidence supporting a causal relationship between endurance training and brain plasticity. Over the two-year follow-up, the sedentary control group exhibited no significant changes in brain structure or function, effectively ruling out maturation effects or spontaneous neural changes unrelated to training. Conversely, the endurance athletes demonstrated training-dependent neuroplasticity adaptations, with moderate-level athletes exhibiting the most extensive structural and functional alterations, including changes in the prefrontal cortex, cerebellum, insula, and basal ganglia. These observed adaptations directly corresponded to their cumulative endurance training volume and intensity, further supporting the conclusion that endurance training actively induces neuroplasticity.

### 4.3. Stage-Dependent Brain Plasticity: Maximum Benefit in Moderate-Level Athletes

To systematically investigate how long-term endurance training impacts brain structure and function at different stages of athletic proficiency, the participants were categorized into three distinct groups: high-level endurance runners, moderate-level endurance runners, and sedentary college students. These groups differed primarily in two dimensions: initial endurance capacity and the intensity and frequency of training during the longitudinal follow-up period. At baseline, the high-level athletes included four national first-level runners and two national master-level runners, with an average of 2.6 ± 1.6 years of systematic endurance training experience. Four of these six participants had completed full marathon races (42.195 km) with the best performances ranging from 2 h 40 min to 3 h. The moderate-level group, though lacking formal athletic rankings, had an average of 5.8 ± 1.8 years of structured training; six out of eight had completed full marathons with the best times ranging from 2 h 53 min to 3 h 51 min. The sedentary controls reported no systematic endurance training and described themselves as sedentary at baseline.

The training load and intensity varied considerably across the groups during the two-year follow-up. The high-level athletes participated in professional training with rigorous management, maintaining high training volumes (5–7 sessions per week, each lasting 1.5–2.5 h). Notably, two athletes progressed from the national first level to master level during this period. The moderate-level athletes engaged in structured, albeit non-professional training, demonstrating considerable adherence, with the frequency ranging from three to ten sessions per week and each session lasting 1–1.5 h. Two participants experienced temporary reductions in training intensity due to injury or other factors but still maintained regular running routines. The sedentary control group remained physically inactive and reported no systematic endurance training.

Strikingly, our longitudinal neuroimaging data revealed the greatest brain plasticity benefits among the moderate-level endurance runners, who exhibited extensive structural and functional reorganization across multiple brain regions. This stage-dependent plasticity highlights two important characteristics. First, neuroplasticity changes critically depend on the optimal training intensity and volume. Insufficient or excessive training beyond adaptive thresholds results in negligible neural adaptations. Second, the endurance level itself is critical: moderate-level athletes demonstrated substantial neural adaptations following two years of training, whereas the participants at both extremes (high-level runners and sedentary controls) showed comparatively fewer changes. The high-level athletes, likely due to previously established neural adaptations, exhibited relatively stable brain structure and function despite high-intensity training. The only significant longitudinal alteration in this group was increased DC in the DLPFC, suggesting subtle neural optimization rather than widespread reorganization.

In conclusion, the moderate-level training stage appears optimal for eliciting maximal brain plasticity benefits, whereas high-level athletes primarily maintain established neural adaptations. This novel insight underscores the importance of personalized training strategies aligned with individual proficiency stages to optimize brain health and performance outcomes. While the participants had prior training history (high-level group: 5.8 ± 1.8 years; moderate-level group: 2.6 ± 1.6 years), the observed plasticity may reflect adaptations to intensified training during the study period. This was evidenced by minimal changes in the high-level athletes despite their advanced baseline, suggesting threshold effects in neural adaptation.

### 4.4. Limitations and Future Directions

Several limitations of the present study warrant consideration. First, the sample size was relatively small, with only ten participants per group, which may reduce the statistical power and limit the generalizability of the findings. Additionally, given the limited sample size, we did not apply a repeated-measures ANOVA or mixed-effects model to assess group × time interactions. Such approaches would allow for a more integrated analysis of both within- and between-group differences. Future research with larger cohorts should consider these statistical methods to validate and extend the current results. In addition, there was an imbalance in the gender ratio across the groups, which may have influenced the observed brain plasticity patterns. Future work with larger and more balanced samples will be needed to control for sex effects and to assess potential sex–training interactions. Second, although this study focused on neuroplasticity outcomes, it did not include concurrent assessments of aerobic fitness or cognitive performance. As a result, we were unable to directly link the observed brain changes to improvements in endurance capacity or cognitive functioning. Incorporating behavioral, physiological, and psychological evaluations in future longitudinal designs would offer a more comprehensive understanding of training-induced brain adaptations. Third, although established MRI metrics were used, we did not acquire test–retest reliability data within our cohort. Future studies should include short-interval rescans to quantify the measurement error thresholds for observed plasticity. Fourth, the absence of intermediate scanning time points is another limitation. Since the study was conducted between 2019 and 2021, the planned interim measurements were disrupted by the COVID-19 pandemic. As a result, we were unable to examine the temporal dynamics of plasticity across training stages. Future studies should consider multi-time point follow-ups to capture potential non-linear trajectories and critical periods of brain plasticity during prolonged endurance training.

### 4.5. Conclusions

Our study results provide a speculative basis that long-term endurance training induces brain plasticity. By tracking athletes at different training stages, our longitudinal data suggest that endurance training induces stage-dependent plasticity, with the maximal effects observed at moderate training levels, while the high-level athletes exhibited stability and the controls showed no change. These findings highlight the stage-dependent nature of neuroplasticity and support targeted endurance training as an effective strategy to enhance brain health.

## Figures and Tables

**Table 1:** Participant demographics.

Variable	High-Level Group	Moderate-Level Group	Control Group
**Baseline**			
Number	n = 10	n = 10	n = 10
Height (cm)	173.6 ± 8.3	171.9 ± 6.4	166.9 ± 7.6
Body mass (kg)	61.7 ± 8.9	64.4 ± 7.8	56.5 ± 9.4
BMI (kg/m^2^)	20.4 ± 1.3	21.7 ± 1.8	20.2 ± 2.1
Years of training	6.3 ± 1.6	2.6 ± 1.0	0.0 ± 0.0
Academic level	Undergraduate	Undergraduate	Undergraduate
Dominant hand	Right		
**Follow-up**			
Number	n = 6	n = 8	n = 8
Height (cm)	171.3 ± 9.9 (n = 6)	171.9 ± 7.0 (n = 8)	163.6 ± 3.3 (n = 8)
Body mass (kg)	59.2 ± 10.0	65.5 ± 8.0	52.5 ± 4.6
BMI (kg/m^2^)	20.0 ± 1.4	22.1 ± 1.7	19.6 ± 1.8
Years of training	5.8 ± 1.8	2.6 ± 1.1	0.0 ± 0.0
Academic level	Undergraduate	Undergraduate	Undergraduate
Dominant hand	Right		

Baseline: Demographic data at baseline testing; Follow-up: Demographic data from the 2-year follow-up assessment.

**Table 2:** Brain plasticity in the high-level endurance group: significant clusters showing longitudinal changes in GMV, DC, and fALFF.

Location	Hemisphere	FWE *p*	Cluster Size	Peak Coordination x	Peak Coordination y	Peak Coordination z	Peak T
GMV							
No significant findings							
fALFF							
No significant findings							
DC							
DLPFC	L	0.002	223	−21	51	18	14.090

Reported activation peaks were obtained from whole-brain voxel-wise analyses with cluster-level family-wise error (FWE) correction at *p* < 0.001; no ROIs were pre-selected. Coordinates are reported in MNI space. Location: anatomical label; Hemisphere: left (L) or right (R); FWE *p*: *p*-value after family-wise error correction at the cluster level; Cluster Size: number of voxels in the significant cluster; Peak Coordination (x, y, z): MNI coordinates of the peak voxel within the cluster; Peak T: t-statistic value for the peak voxel within the cluster. GMV: gray matter volume; fALFF: fractional amplitude of low-frequency fluctuations; DC: degree centrality; DLPFC: left dorsolateral prefrontal cortex.

**Table 3:** Brain plasticity in the moderate-level running group: significant clusters showing longitudinal changes in GMV, DC, and fALFF.

Location	Hemisphere	FWE *p*	Cluster Size	Peak Coordination x	Peak Coordination y	Peak Coordination z	Peak T
GMV							
DLPFC	R	0.001	12	44	48	17	9.996
DLPFC	R	0.003	11	33	53	20	8.840
DLPFC	R	0.016	9	35	45	30	7.226
DLPFC	L	0.016	9	−29	26	51	11.323
Premotor cortex	R	<0.001	65	24	−4	57	9.565
Premotor cortex	L	0.001	12	−33	5	56	7.296
Premotor cortex	L	0.016	9	−18	23	57	8.565
Premotor cortex	L	0.003	11	−32	20	50	9.433
Premotor cortex	R	0.001	12	42	27	42	8.686
Premotor cortex	R	0.038	8	27	11	54	7.593
Premotor cortex	R	0.003	11	24	17	62	7.645
Precentral gyrus	R	0.038	8	54	−23	35	8.977
Postcentral gyrus	R	<0.001	23	41	−23	51	10.981
Medial frontal lobe	L	0.038	8	−6	51	−8	7.686
Medial frontal lobe	L	0.001	13	−11	26	57	7.616
Medial frontal lobe	L	0.038	8	−3	42	9	7.391
Medial frontal lobe	R	<0.001	14	6	44	44	9.397
Medial frontal lobe	R	0.016	9	9	3	71	7.885
Insular	R	0.003	11	38	−7	14	13.668
Putamen	R	0.003	11	17	0	18	12.046
Putamen	R	0.007	10	33	5	1	8.347
Frontoparietal occipitotemporal junction	R	0.016	9	42	−29	12	11.791
Frontoparietal occipitotemporal junction	R	0.016	9	51	−29	24	6.590
Frontoparietal occipitotemporal junction	R	0.016	9	30	3	6	6.351
Posterior lobe of cerebellum	R	<0.001	217	20	−72	−53	−14.086
Posterior lobe of cerebellum	L	<0.001	46	−20	−80	−48	−11.766
Posterior lobe of cerebellum	R	<0.001	18	26	−75	−39	−7.571
Posterior lobe of cerebellum	L	0.038	8	−15	−72	−53	−7.337
fALFF							
Medial frontal lobe	R	<0.001	41	42	6	33	10.329
DC							
DLPFC	R	<0.001	1201	42	21	27	13.763
DLPFC	L	<0.001	541	−39	9	39	9.697
Temporal lobe	R	0.042	129	66	0	−9	16.237
Thalamus	L	<0.001	4008	−3	−27	9	28.966

Reported activation peaks were obtained from whole-brain voxel-wise analyses with cluster-level family-wise error (FWE) correction at *p* < 0.001; no ROIs were pre-selected. Coordinates are reported in MNI space. Multiple entries with the same anatomical label indicate distinct significant clusters located in different hemispheres or subregions, each with unique peak coordinates and spatial extents. Location: anatomical label; Hemisphere: left (L) or right (R); FWE *p*: *p*-value after family-wise error correction at the cluster level; Cluster Size: number of voxels in the significant cluster; Peak Coordination (x, y, z): MNI coordinates of the peak voxel within the cluster; Peak T: t-statistic value for the peak voxel within the cluster. GMV: gray matter volume; fALFF: fractional amplitude of low-frequency fluctuations; DC: degree centrality; DLPFC: left dorsolateral prefrontal cortex.

## Data Availability

Due to the involvement of elite athletes and the sensitivity of their fMRI data, the original datasets cannot be made publicly available in order to protect participants’ privacy. However, anonymized data from non-elite participants can be obtained from the corresponding author upon reasonable request

## Abbreviations

GMV	gray matter volume
FC	functional connectivity
DLPFC	left dorsolateral prefrontal cortex
fALFF	fractional amplitude of low-frequency fluctuations
DC	degree centrality
EPI	echo-planar imaging
TR	repetition time
TE	echo time
FWHM	full width at half maximum
FOV	field of view
FWE	family-wise error
DMN	default mode network
CON	cingulo-opercular network
mPFC	medial prefrontal cortex
BDNF	brain-derived neurotrophic factor
NFA	National First-Class Athlete
MA	Master Athlete

## References

[1] Herting M.M., Colby J.B., Sowell E.R., Nagel B.J. (2014). White matter connectivity and aerobic fitness in male adolescents. Dev. Cogn. Neurosci..

[2] Raichlen D.A., Bharadwaj P.K., Fitzhugh M.C., Haws K.A., Torre G.-A., Trouard T.P., Alexander G.E. (2016). Differences in resting state functional connectivity between young adult endurance athletes and healthy controls. Front. Hum. Neurosci..

[3] Zhang K., Cao C., Wang Y., Zhang D. (2024). Brain structure and function differences across varying levels of endurance training: A cross-sectional study. Front. Hum. Neurosci..

[4] Hedeker D., Gibbons R.D. (2006). Longitudinal Data Analysis.

[5] Ridgway G., Ashburner J., Penny W. (2013). Modelling Longitudinal Structural Change from Serial MRI.

[6] Qu H., Tang H., Wang L., Wang W., Zhao Y., Chen A., Hu C. (2025). Effects on brain structural and functional in deaf children after aerobic exercise training: A pilot cluster randomized controlled study. Int. J. Neurosci..

[7] Colcombe S.J., Erickson K.I., Scalf P.E., Kim J.S., Prakash R., McAuley E., Elavsky S., Marquez D.X., Hu L., Kramer A.F. (2006). Aerobic exercise training increases brain volume in aging humans. J. Gerontol. A Biol. Sci. Med. Sci..

[8] Yogev-Seligmann G., Eisenstein T., Ash E., Giladi N., Sharon H., Nachman S., Bregman N., Kodesh E., Hendler T., Lerner Y. (2021). Neurocognitive plasticity is associated with cardiorespiratory fitness following physical exercise in older adults with amnestic mild cognitive impairment. J. Alzheimers Dis..

[9] El-Sayes J., Harasym D., Turco C.V., Locke M.B., Nelson A.J. (2019). Exercise-Induced Neuroplasticity: A Mechanistic Model and Prospects for Promoting Plasticity. Neuroscientist.

[10] Lindquist K., Guadagnoli M. (2008). Neuroanatomical correlates of motor skill learning: Inferences from neuroimaging to behavior. Adv. Psychol..

[11] Salehi S.K., Tahmasebi F., Talebrokni F.S. (2021). A different look at featured motor learning models: Comparison exam of Gallahue’s, Fitts and Posner’s and Ann Gentile’s motor learning models. Mov. Sport Sci..

[12] Di Domenico S.I., Rodrigo A.H., Ayaz H., Fournier M.A., Ruocco A.C. (2015). Decision-making conflict and the neural efficiency hypothesis of intelligence: A functional near-infrared spectroscopy investigation. Neuroimage.

[13] Micheloyannis S., Pachou E., Stam C.J., Vourkas M., Erimaki S., Tsirka V. (2006). Using graph theoretical analysis of multi channel EEG to evaluate the neural efficiency hypothesis. Neurosci. Lett..

[14] Hou H.-Y., Jia X.-Z., Wang P., Zhang J.-X., Huang S., Li H.-J. (2019). Intrinsic resting-state activity in older adults with video game experience. Front. Aging Neurosci..

[15] Zuo X.N., Di Martino A., Kelly C., Shehzad Z.E., Gee D.G., Klein D.F., Castellanos F.X., Biswal B.B., Milham M.P. (2010). The oscillating brain: Complex and reliable. Neuroimage.

[16] Braun U., Plichta M.M., Esslinger C., Sauer C., Haddad L., Grimm O., Mier D., Mohnke S., Heinz A., Erk S. (2012). Test-retest reliability of resting-state connectivity network characteristics using fMRI and graph theoretical measures. Neuroimage.

[17] McDermott K.B., Buckner R.L., Petersen S.E., Kelley W.M., Sanders A.L. (1999). Set-and code-specific activation in the frontal cortex: An fMRI study of encoding and retrieval of faces and words. J. Cogn. Neurosci..

[18] Lepage M., Ghaffar O., Nyberg L., Tulving E. (2000). Prefrontal cortex and episodic memory retrieval mode. Proc. Natl. Acad. Sci. USA.

[19] Cabeza R., Dolcos F., Graham R., Nyberg L. (2002). Similarities and differences in the neural correlates of episodic memory retrieval and working memory. Neuroimage.

[20] Schafer R.J., Moore T. (2011). Selective attention from voluntary control of neurons in prefrontal cortex. Science.

[21] Jäncke L., Koeneke S., Hoppe A., Rominger C., Hänggi J. (2009). The architecture of the golfer’s brain. PLoS ONE.

[22] Rovio S., Spulber G., Nieminen L.J., Niskanen E., Winblad B., Tuomilehto J., Nissinen A., Soininen H., Kivipelto M. (2010). The effect of midlife physical activity on structural brain changes in the elderly. Neurobiol. Aging.

[23] Wood K.N., Nikolov R., Shoemaker J.K. (2016). Impact of long-term endurance training vs. guideline-based physical activity on brain structure in healthy aging. Front. Aging Neurosci..

[24] Ji L., Zhang H., Potter G.G., Zang Y.-F., Steffens D.C., Guo H., Wang L. (2017). Multiple neuroimaging measures for examining exercise-induced neuroplasticity in older adults: A quasi-experimental study. Front. Aging Neurosci..

[25] Alvarez J.A., Emory E. (2006). Executive function and the frontal lobes: A meta-analytic review. Neuropsychol. Rev..

[26] Eckert M.A., Menon V., Walczak A., Ahlstrom J., Denslow S., Horwitz A., Dubno J.R. (2009). At the heart of the ventral attention system: The right anterior insula. Hum. Brain Mapp..

[27] Seeley W.W., Menon V., Schatzberg A.F., Keller J., Glover G.H., Kenna H., Reiss A.L., Greicius M.D. (2007). Dissociable intrinsic connectivity networks for salience processing and executive control. J. Neurosci..

[28] Chan R.C., Shum D., Toulopoulou T., Chen E.Y. (2008). Assessment of executive functions: Review of instruments and identification of critical issues. Arch. Clin. Neuropsychol..

[29] Bezzola L., Mérillat S., Gaser C., Jäncke L. (2011). Training-induced neural plasticity in golf novices. J. Neurosci..

[30] Milton J., Solodkin A., Hluštík P., Small S.L. (2007). The mind of expert motor performance is cool and focused. Neuroimage.

[31] Wei G., Luo J., Li Y. (2009). Brain structure in diving players on MR imaging studied with voxel-based morphometry. Prog. Nat. Sci..

[32] Hänggi J., Koeneke S., Bezzola L., Jäncke L. (2010). Structural neuroplasticity in the sensorimotor network of professional female ballet dancers. Hum. Brain Mapp..

[33] Mantini D., Vanduffel W. (2013). Emerging roles of the brain’s default network. Neuroscientist.

[34] Buckner R.L., Andrews-Hanna J.R., Schacter D.L. (2008). The brain’s default network: Anatomy, function, and relevance to disease. Ann. N. Y. Acad. Sci..

[35] Fox M.D., Snyder A.Z., Vincent J.L., Corbetta M., Van Essen D.C., Raichle M.E. (2005). The human brain is intrinsically organized into dynamic, anticorrelated functional networks. Proc. Natl. Acad. Sci. USA.

[36] Raichle M.E., MacLeod A.M., Snyder A.Z., Powers W.J., Gusnard D.A., Shulman G.L. (2001). A default mode of brain function. Proc. Natl. Acad. Sci. USA.

[37] Martinelli P., Sperduti M., Piolino P. (2013). Neural substrates of the self-memory system: New insights from a meta-analysis. Hum. Brain Mapp..

[38] Dörfel D., Werner A., Schaefer M., Von Kummer R., Karl A. (2009). Distinct brain networks in recognition memory share a defined region in the precuneus. Eur. J. Neurosci..

[39] Wang B., Fan Y., Lu M., Li S., Song Z., Peng X., Zhang R., Lin Q., He Y., Wang J. (2013). Brain anatomical networks in world class gymnasts: A DTI tractography study. Neuroimage.

[40] Vaina L.M., Solomon J., Chowdhury S., Sinha P., Belliveau J.W. (2001). Functional neuroanatomy of biological motion perception in humans. Proc. Natl. Acad. Sci. USA.

[41] Park I.S., Han J.W., Lee K.J., Lee N.J., Lee W.T., Park K.A., Rhyu I.J. (2006). Evaluation of morphological plasticity in the cerebella of basketball players with MRI. J. Korean Med. Sci..

[42] Vrabel M. (2015). Preferred reporting items for systematic reviews and meta-analyses. Oncol. Nurs. Forum.

[43] Van Den Heuvel M.P., Pol H.E.H. (2010). Exploring the brain network: A review on resting-state fMRI functional connectivity. Eur. Neuropsychopharmacol..

[44] Phinyomark A., Ibanez-Marcelo E., Petri G. (2017). Resting-state fMRI functional connectivity: Big data preprocessing pipelines and topological data analysis. IEEE Trans. Big Data.

[45] Fu M., Yu X., Lu J., Zuo Y. (2012). Repetitive motor learning induces coordinated formation of clustered dendritic spines in vivo. Nature.

[46] Cole M.W., Reynolds J.R., Power J.D., Repovs G., Anticevic A., Braver T.S. (2013). Multi-task connectivity reveals flexible hubs for adaptive task control. Nat. Neurosci..

[47] Cross E.S., Hamilton A.F.d.C., Grafton S.T. (2006). Building a motor simulation de novo: Observation of dance by dancers. Neuroimage.

[48] Gondoh Y., Sensui H., Kinomura S., Fukuda H., Fujimoto T., Masud M., Nagamatsu T., Tamaki H., Takekura H. (2009). Effects of aerobic exercise training on brain structure and psychological well-being in young adults. J. Sports Med. Phys. Fit..

[49] Kelly A.C., Garavan H. (2005). Human functional neuroimaging of brain changes associated with practice. Cereb. Cortex.

[50] Uysal N., Tugyan K., Kayatekin B.M., Acikgoz O., Bagriyanik H.A., Gonenc S., Ozdemir D., Aksu I., Topcu A., Semin I. (2005). The effects of regular aerobic exercise in adolescent period on hippocampal neuron density, apoptosis and spatial memory. Neurosci. Lett..

[51] Firth J., Stubbs B., Vancampfort D., Schuch F., Lagopoulos J., Rosenbaum S., Ward P.B. (2018). Effect of aerobic exercise on hippocampal volume in humans: A systematic review and meta-analysis. Neuroimage.

[52] Cotman C.W., Berchtold N.C., Christie L.-A. (2007). Exercise builds brain health: Key roles of growth factor cascades and inflammation. Trends Neurosci..

[53] Aguiar A.S., Speck A.E., Prediger R.D., Kapczinski F., Pinho R.A. (2008). Downhill training upregulates mice hippocampal and striatal brain-derived neurotrophic factor levels. J. Neural Transm..

[54] Esteban-Cornejo I., Cadenas-Sanchez C., Contreras-Rodriguez O., Verdejo-Roman J., Mora-Gonzalez J., Migueles J.H., Henriksson P., Davis C.L., Verdejo-Garcia A., Catena A. (2017). A whole brain volumetric approach in overweight/obese children: Examining the association with different physical fitness components and academic performance. The ActiveBrains project. Neuroimage.

[55] Yook J.S., Rakwal R., Shibato J., Takahashi K., Koizumi H., Shima T., Ikemoto M.J., Oharomari L.K., McEwen B.S., Soya H. (2019). Leptin in hippocampus mediates benefits of mild exercise by an antioxidant on neurogenesis and memory. Proc. Natl. Acad. Sci. USA.

[56] So J.H., Huang C., Ge M., Cai G., Zhang L., Lu Y., Mu Y. (2017). Intense Exercise Promotes Adult Hippocampal Neurogenesis But Not Spatial Discrimination. Front. Cell. Neurosci..

